# Changes in Deoxyribonucleic Acid Methylation Contribute to the Pathophysiology of Multiple Sclerosis

**DOI:** 10.3389/fgene.2019.01138

**Published:** 2019-11-12

**Authors:** Naiara Celarain, Jordi Tomas-Roig

**Affiliations:** Girona Neuroimmunology and Multiple Sclerosis Unit (UNIEM), Dr. Josep Trueta University Hospital, Girona Biomedical Research Institute (IDIBGI), Girona, Spain

**Keywords:** multiple sclerosis, deoxyribonucleic acid methylation, immune cells, central nervous system, deoxyribonucleic acid damage

## Abstract

Multiple sclerosis (MS) is an autoimmune disease of the central nervous system characterized by loss of coordination, weakness, dysfunctions in bladder capacity, bowel movement, and cognitive impairment. Thus, the disease leads to a significant socioeconomic burden. In the pathophysiology of the disease, both genetic and environmental risk factors are involved. Gene x environment interaction is modulated by epigenetic mechanisms. Epigenetics refers to a sophisticated system that regulates gene expression with no changes in the DNA sequence. The most studied epigenetic mechanism is the DNA methylation. In this review, we summarize the data available from the current literature by grouping sets of differentially methylated genes in distinct biological categories: the immune system including innate and adaptive response, the DNA damage, and the central nervous system.

## Introduction

Multiple sclerosis (MS) is a potentially disabling central nervous system (CNS) disease characterized by inflammation, demyelination, and axonal degeneration. The pathophysiological mechanisms involved in this autoimmune disease differ between patients. In MS pathogenesis, antigen-presenting cells (APCs) stimulate CD4+ T cells in the periphery, favoring their differentiation into CD4+ T helper (Th) cells. Upon their activation, these immune cells cross the blood–brain barrier (BBB) and migrate into the brain, where they are reactivated by resident APCs. Proinflammatory cytokines and chemokines are released by reactivated CD4+ T cells, while infiltrated cytotoxic CD8+ T cells impair neuronal integrity. In parallel, plasma B cells release antibodies against self myelin epitopes, contributing to demyelination (Ghasemi et al., 2017) (**Figure S1**).

**Figure 1:**
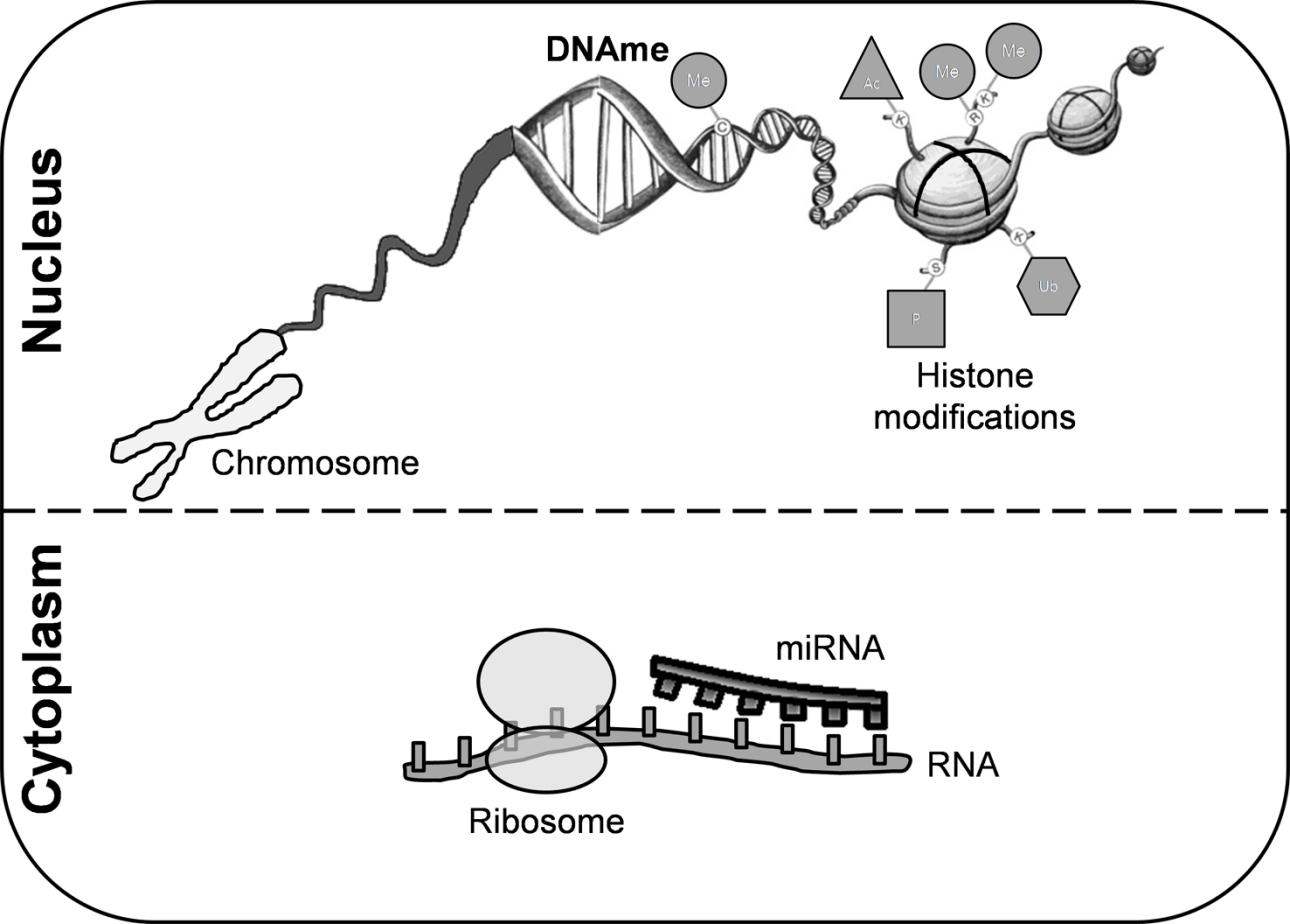
Epigenetic modifications. These mechanisms are crucial for regulating gene transcription and chromatin architecture. Among them, we can highlight histone modifications, DNA methylation, and microRNAs. Covalent modifications of histones include acetylation, phosphorylation, sumoylation, ubiquitination, and methylation. DNA methylation is the most common epigenetic mechanism that occurs mainly in enriched CG dinucleotides regions in somatic cells. miRNAs are small non-coding RNA molecules that participate in RNA silencing. DNAme, DNA methylation; miRNA, microRNA; Me, methylation; Ub, ubiquitination; Ac, acetylation; P, phosphorylation.

An individual’s genetic background and exposure to environmental factors confer risk of developing MS through epigenetic modifications (**Figure 1**). DNA methylation (DNAme) is the most common epigenetic mechanism in somatic cells. This process occurs mainly in regulatory and promoter regions, where cytosine-guanine dinucleotides are markedly present (Weber et al., 2007). Although the precise mechanism of action of DNAme in MS remains to be fully elucidated, several studies have reported differentially methylated regions in either lymphocytes or peripheral blood mononuclear cells (PBMCs) and in *post-mortem* brain tissue (**Table S1**).

In this study, sets of differentially methylated genes described in the relevant literature are compared using Venn diagrams in order to determine the common, overlapping genes (**Table 1**). Although the studies included in this review have used a variety of methods, target samples, and subjects at different stages of the disease with distinct demographic characteristics that may contribute to DNAme heterogeneity, we assume that the common results reported at cell type level by different case studies could potentially explain in part MS pathophysiology. These results are summarized below.

**Table 1:** Overlapped genes obtained after Venn diagram analysis.

Gene symbol	Reference	Methylation status	Comparison	Target sample	Biological function
**AHRR**	Chomyk et al., 2017	Hypo	Within MS patients	NAWM; demyelinated hippocampus	Homeostasis of the immune system
	Graves et al., 2014	Hypo	RRMS *vs.* CTR	CD4+ T cells	
	Marabita et al., 2017	Hypo	Smoker MS *vs.* non-smoker MS	PMBCs	
**RASA3**	Chomyk et al., 2017	Hypo	Within MS patients	NAWM; demyelinated hippocampus	Inhibition of pathogenic Th17 cells
	Huynh et al., 2014	Hyper	MS *vs.* CTR	NAWM	
	Kulakova et al., 2016	Hyper	RRMS *vs.* SPMS	PMBCs	
**MORN1**	Graves et al., 2014	Hyper	RRMS *vs.* CTR	CD4+ T cells	Regulation of calcium homeostasis
	Maltby et al., 2015	Hyper	MS *vs.* CTR	CD8+ T cells	
**KIF25**	Chomyk et al., 2017	Hypo	Within MS patients	NAWM; demyelinated hippocampus	Motor protein involved in trafficking of vesicles, organelles, and proteins through the cytoskeleton
	Graves et al., 2014	Hypo	RRMS *vs.* CTR	CD4+ T cells	
**TGFBI**	Chomyk et al., 2017	Hypo	Within MS patients	NAWM; demyelinated hippocampus	Participate in calcium signaling and inflammation process
	Graves et al., 2014	Hyper	RRMS *vs.* CTR	CD4+ T cells	
**USP35**	Graves et al., 2014	Hypo	RRMS *vs.* CTR	CD4+ T cells	Deubiquitinating enzyme involved in type I interferon signaling
	Kulakova et al., 2016	Hyper	RRMS *vs.* SPMS	PMBCs	
**MICB**	Graves et al., 2014	Hypo	RRMS *vs.* CTR	CD4+ T cells	Involved in innate immune system regulation
	Huynh et al., 2014	Hypo	MS *vs.* CTR	NAWM	
**IGSF9B**	Chomyk et al., 2017	Hyper	Within MS patients	NAWM; demyelinated hippocampus	Cell adhesion molecule involved in GABAergic circuits
	Kulakova et al., 2016	Hyper	PPMS *vs.* CTR	PMBCs	
**PSD3**	Bos et al., 2015	Hypo	RRMS *vs.* CTR	CD8+ T cells	Control of neurite formation, spine density, trafficking of synaptic vesicles
	Chomyk et al., 2017	Hypo	Within MS patients	NAWM; demyelinated hippocampus	
**HLA-F**	Huynh et al., 2014	Hypo	MS *vs.* CTR	NAWM	Regulation of immune response through antigen-processing mechanism
	Kulakova et al., 2016	Hypo	PPMS *vs.* CTR	PMBCs	
**GNAS**	Huynh et al., 2014	Hypo	MS *vs.* CTR	NAWM	Involved in Th17 activation and autoimmunity
	Kulakova et al., 2016	Hypo	RRMS *vs.* CTR	PMBCs	
**ATP11A**	Huynh et al., 2014	Hyper	MS *vs.* CTR	NAWM	Possess an anti-inflammatory activity through internalization of macrophage TLR-4
	Kulakova et al., 2016	Hypo	RRMS *vs.* CTR	PMBCs	
**HOXC4**	Huynh et al., 2014	Hypo	MS *vs.* CTR	NAWM	Involved in vasculature pathways, nucleosome organization, and autoimmune disorders
	Kulakova et al., 2016	Hypo	RRMS *vs.* CTR	PMBCs	
**RARA**	Huynh et al., 2014	Hypo	MS *vs.* CTR	NAWM	Regulation of development, differentiation, apoptosis, granulopoiesis, and transcription of clock genes
	Marabita et al., 2017	Hypo	Smoker MS *vs.* non-smoker MS	PMBCs	
**PTPRN2**	Bos et al., 2015	Hypo	RRMS *vs.* CTR	CD8+ T cells	Proliferation of regulatory T cells
	Huynh et al., 2014	Hyper	MS *vs.* CTR	NAWM	
**CDH1**	Huynh et al., 2014	Hyper	MS *vs.* CTR	NAWM	Cell adhesion protein involved in synaptogenesis
	Liggett et al., 2010	Hyper	RRMS (r) *vs.* CTR RRMS (r) *vs.* RRMS (e)	cfpDNA	
**LINE-1**	Dunaeva et al., 2018	Hyper	RRMS *vs.* CTR	cfDNA (serum)	Retrotransposons
	Pinto-Medel et al., 2017	Hyper	MS naïve *vs.* MS IFN-β 1 year *vs.* CTR	PMBCs	
**RUNX3**	Huynh et al., 2014	Hypo	MS *vs.* CTR	NAWM	Coordination of DC, T, and NK cell differentiation
	Sokratous et al., 2018	Hyper	RRMS (e), RRMS (r) *vs.* CTR	Whole blood	
**CDKN2A**	Liggett et al., 2010	Hyper	RRMS (r) *vs.* CTR	cfpDNA	Regulation of cell cycle
	Sokratous et al., 2018	Hyper	RRMS (e), RRMS (r) *vs.* CTR	Whole blood	
**SOCS1**	Liggett et al., 2010	Hyper	RRMS (r) *vs.* CTR	cfpDNA	Regulation of proinflammatory cytokines release
	Sokratous et al., 2018	Hyper	RRMS (e) RRMS (r) *vs.* CTR	Whole blood	

NAWM, normal appearing white matter; PBMCs, peripheral blood mononuclear cells; cfpDNA, cell-free plasma DNA; cfDNA, circulating free DNA.

### The Immune System

The homeostasis of the immune system is modulated by the aryl hydrocarbon receptor (AHR). AHR activity is negatively regulated by the encoded protein for the aryl hydrocarbon receptor repressor (AHRR). MS patients showed lower expression levels of circulating AHR than their matched controls (Neavin et al., 2018). In line with these findings, lower DNAme levels for AHRR have been measured in demyelinated hippocampi (Chomyk et al., 2017), CD4+ T cells (Graves et al., 2014), and PBMCs (Marabita et al., 2017) of MS patients. This suggests that immune differentiation as well as the clinical course are compromised in MS (Neavin et al., 2018). Furthermore, it is widely accepted that the major histocompatibility complex (MHC) plays a key role in the genetic susceptibility to MS. Two polymorphic genes, termed MHC class I chain-related gene A (MICA) and MHC class I chain-related gene B (MICB), are located within the MHC class I region. These molecules interact with specific receptors constitutively expressed in natural killer (NK) and T cells. The expression of MICB proteins in circulating PBMCs stimulates autoreactive T cells and favors MS progression (Abediankenari et al., 2011). Similarly, Fernandez-Morera et al. (2008) found that the MICB*004 allele was significantly higher in MS patients than their matched controls. MS patients displayed lower DNAme levels for MICB compared to controls (Graves et al., 2014; Huynh et al., 2014), in agreement with Abediankenari et al. (2011) and Fernandez-Morera et al. (2008). Runt-related transcription factor 3 (RUNX3) is expressed in dendritic cells (DCs), as well as T and NK cells, regulating their differentiation. In contrast, cyclin-dependent kinase inhibitor 2A (CDKN2A) controls the cell cycle, and it is abundantly expressed in oligodendrocytes and CD4+ and CD8+ T cells. In PBMCs collected from MS patients, both genes were underexpressed, which indicates a misbalance in CD4/CD8 T cell differentiation (Parnell et al., 2014). In line with these findings, elevated levels of DNAme for RUNX3 and CDKN2A were found in MS patients compared to controls (Liggett et al., 2010; Sokratous et al., 2018).

### Innate Immune Response

In DCs, high levels of cyclic adenosine monophosphate (cAMP) activate Th17 response through the stimulation of guanine nucleotide-binding protein, alpha stimulating (GNAS). High levels of activated Th17 cells are associated with autoimmunity (Lee et al., 2015). In line with these findings, other studies have revealed a lower number of DNAme groups to GNAS (Huynh et al., 2014; Kulakova et al., 2016) and consequently, an overactivation of Th17 cells. It is well-known that the recognition of CNS self-epitopes by monocytes facilitates type I interferon (IFN) release and thus, autoimmunity. IFN signaling is regulated by ubiquitination mechanisms. Ubiquitin-specific peptidase 35 (USP35), a member of the deubiquitinating enzyme family, reverses the process of ubiquitination and confers neuroprotection. During an inflammatory response, USP35 is underexpressed in monocytes. The expression of this deubiquitinating enzyme is higher when the inflammation is mitigated (Liu et al., 2018). Thus, we can speculate that a high number of DNAme groups to USP35 might occur in parallel with a relapsing MS course (Kulakova et al., 2016), while a hypomethylated pattern could favor a remitting course for the disease (Graves et al., 2014). In macrophages, the group of phosphatidylserines, a canonical substrate for ATPase phospholipid transporting 11A (ATP11A), is charged negatively and participates in the internalization of toll-like receptor 4 (TLR4). The internalization of TLR4 is essential to restricting long-term inflammatory responses. Deletion of ATP11A in humans causes an exacerbated inflammatory response (van der Mark et al., 2017). The addition of DNAme groups to ATP11A, which Huynh et al. (2014) described in the CNS of MS patients, might result in lower protein content and, consequently, chronic inflammation. However, Kulakova et al. (2016) found an opposite effect, indicating that the expression of ATP11A was presumably higher in MS patients than controls. This discrepancy might suggest that the study of DNAme derived from PBMCs (Kulakova et al., 2016) was conducted during the remitting course of MS, when no signs of inflammation were present.

### Adaptive Immune Response

CD4+ T cells proliferate into effector T cells in order to provide the most effective response to maintain immune homeostasis. Among CD4+ T cells, the Th17 subset regulates the immune response against autoimmunity. The generation of pathogenic Th17 (pTh17) cells is associated with an upregulation of RAS p21 protein activator 3 (RASA3). In contrast, reduced expression of RASA3 suppresses pTh17 cell generation *via* enhanced interleukin 4 synthesis (Wu et al., 2018). An elevated number of methyl groups for RASA3 was measured in normal-appearing white matter (NAWM) (Huynh et al., 2014) and PBMCs (Kulakova et al., 2016), presumably due to the fact that MS patients displayed no radiological disease activity and absence of pTh17 cells. In contrast, Chomyk et al. (2017) demonstrated a hypomethylated RASA3 pattern in the hippocampus during ongoing demyelination. Therefore, the migration of pathogenic lymphocytes into the MS brain originates in the destruction of myelin sheath and axonal degeneration through elevated levels of RASA3 (Wu et al., 2018). The activation of homeobox C4 (HOXC4) promotes the proliferation and differentiation of B cells (Park et al., 2013). High mRNA transcription rates for HOXC4 are associated with dysfunctions in vasculature pathways and nucleosome organization (Marchetti et al., 2018) and have also been observed in autoimmune disorders (Park et al., 2013). A lower number of methylated groups to HOXC4 would result in elevated protein levels, contributing to autoimmunity (Huynh et al., 2014; Kulakova et al., 2016). The proliferation of regulatory T cells is achieved through the interaction of protein tyrosine phosphatase receptor type N2 (PTPRN2) with SMAD family member 3 (SMAD3) (Lee et al., 2016). A hypomethylated pattern of PTPRN2 has been described in T cells collected from MS patients (Bos et al., 2015). Thus, high levels of PTPRN2 might potentiate the proliferation of regulatory T cells. Interestingly, transforming growth factor, beta-induced (TGFBI) prevents autoimmunity by promoting T cell activation through Ca^2+^-calcineurin signaling (Graca, 2007). The addition of methyl groups to TGFBI in CD4+ T cells derived from MS patients (Graves et al., 2014) might prevent autoimmune reactivity favoring Ca^2+^-calcineurin signaling and T cell activation (Graca, 2007). Cytokines participate in the differentiation, maturation, and survival of immune cells. Suppressor of cytokine signaling 1 (SOCS1) regulates specifically the release of proinflammatory cytokines in MS. Under pathophysiological conditions, a significant reduction of SOCS1 has been measured in parallel with the synthesis of proinflammatory cytokines in MS (Toghi et al., 2017). In agreement with Toghi et al. (2017), a substantial number of DNAme groups to SOCS1 might result in an acute inflammatory response, as has been reported in the context of MS (Liggett et al., 2010; Sokratous et al., 2018).

### Deoxyribonucleic Acid Damage

The endoplasmic reticulum (ER), along with the plasma membrane (PM) junctions, is essential for Ca^2+^ homeostasis. ER Ca^2+^ depletion potentiates the inflammatory response, ER stress and, lastly, DNA damage (Woo et al., 2016). Upon activation of T-cell receptors (TCRs), protein junctions containing membrane occupation and recognition nexus (MORN) motifs stimulate a Ca^2+^ influx in T cells. Deficiency of protein junctions containing MORN motifs aggravates the store-operated Ca^2+^ entry in T cells and causes DNA damage (Woo et al., 2016). An elevated number of DNAme groups to MORN repeat containing 1 (MORN1) has been reported in both CD4+ and CD8+ T cells collected from MS patients (Graves et al., 2014; Maltby et al., 2015) and might affect T cell viability (Woo et al., 2016). On the other hand, pleckstrin and Sec7 domain-containing 3 (PSD3) are predominantly expressed in the CNS (Schraut et al., 2014), as well as in T cells, macrophages, and neutrophils (Okada et al., 2012). The Sec7 domain of PSD3 protein is a guanine nucleotide exchange factor for small GTPases that contributes to neurite formation, spine density, trafficking of synaptic vesicles, and receptor internalization (Okada et al., 2012). High levels of PSD3 activate Fas-induced apoptosis, while its depletion disrupts cell shape and polarity (Okada et al., 2012). In MS, a hypomethylation of PSD3 has been reported in both CD8+ T cells (Bos et al., 2015) and demyelinated hippocampi (Chomyk et al., 2017). This might suggest that immune homeostasis is unbalanced under these conditions (Okada et al., 2012).

*De novo* telomere addition of long interspersed nuclear elements (LINE-1) into genomic DNA causes genetic defects, alters the regulatory mechanisms and the structural properties of the genome at their insertion place and might lead to genomic remodeling (Furano, 2000). Changes in LINE-1 methylation status have been described in MS patients. Indeed, LINE-1 CpG sites were more frequently methylated in MS patients than in controls (Pinto-Medel et al., 2017; Dunaeva et al., 2018). A hypermethylated DNA pattern for LINE-1 in MS might be related to chromosomal abnormalities and DNA damage (Pinto-Medel et al., 2017).

### The Central Nervous System

Observations of human MS plaques have revealed an increase of protein tyrosine phosphatases (PTPs) during ongoing remyelination (Hendriks et al., 2013). In NAWM derived from MS patients, the addition of DNAme to PTPRN2 (Huynh et al., 2014), a member of the PTP family, indicates that the CNS integrity remains unaltered. An upregulation of TGFBI is involved in CNS repair after brain injury through the inhibition of the inflammatory response, mediated by activated microglia. Therefore, TGFBI acts as a suppressor of microgliosis (Kim and Lee, 2011). Consequently, lower DNAme levels to TGFBI in the demyelinated hippocampus of MS patients might occur in order to counteract the deleterious effect associated with the activation of microglia (Kim and Lee, 2011). Kinesins (KIFs), a family of motor proteins mainly expressed in neurons, immune cells, and oligodendrocytes, are involved in the trafficking of vesicles, organelles, and proteins through the cytoskeleton (Hirokawa and Takemura, 2013). An overexpression of KIF improves axonal transport, while its downregulation leads to neurodegeneration and CNS atrophy (Lo Giudice et al., 2006). Dysregulation of certain KIFs has been postulated to aggravate MS disease (Hares et al., 2014). Lower methylated levels for KIF family member 25 (KIF25) have been measured in the hippocampi (Chomyk et al., 2017) and CD4+ T cells (Graves et al., 2014) of MS patients. Thus, it is plausible that lower DNAme levels could increase the production of KIF motor proteins to compensate the increased CNS molecule transport demand in MS patients. Cell adhesion molecules (CAMs) participate in axon guidance, synaptogenesis, and neuronal regeneration (Hansen and Walmod, 2013). Immunoglobulin superfamily member 9B (IGSF9B) is a member of the CAM family highly expressed in GABAergic interneurons, macrophages/microglia, and astrocytes (Gil-Varea et al., 2018). IGSF9B is also constitutively expressed in circulating monocytes (Tserel et al., 2014); however, its biological function in this cell type remains unclear and thus requires further investigation. Elevated expression of IGSF9B favors the maintenance of inhibitory synapses (Gil-Varea et al., 2018), while low levels result in a selective loss of GABAergic interneurons (Mishra et al., 2014). A prominent loss of GABAergic circuits has been described in MS (Gil-Varea et al., 2018). The findings reported by Chomyk et al. (2017) and Kulakova et al. (2016) suggest that a substantial number of DNAme to IGSF9B could be accompanied by lower transcription rates as well as a selective loss of GABAergic interneurons (Gil-Varea et al., 2018). In MS, HLA class I histocompatibility antigen, alpha chain F (HLA-F) cooperates with the antigen-processing peptide-loading complex, regulating the immune response *via* NK receptors (Alcina et al., 2012). An overexpression of HLA-F protects neurons from astrocyte-mediated neurotoxicity (Song et al., 2016). Thus, it can be hypothesized that a lower number of methyl groups to HLA-F might confer neuroprotection. In this regard, Huynh et al. (2014) found a hypomethylation of HLA-F promoter in NAWM collected from MS patients compared to their controls. It is noteworthy that RUNX3, a transcription factor importantly involved in DC and T and NK cell differentiation, is upregulated in active and chronic MS lesions (Sokratous et al., 2018). Similarly, Huynh et al. (2014) reported a hypomethylated DNA pattern for RUNX3 in NAWM collected from MS patients. This indicates that RUNX3 DNAme status could be used as an inflammatory biomarker even when inflammation is apparently not present in the CNS (Huynh et al., 2014). When retinoic acid (RA) interacts with retinoic acid receptor alpha (RARA), several transcriptional changes affecting the immune system and CNS repair can take place. Indeed, the inflammatory activity observed in MS patients is attenuated upon stimulation of RARA (Kim et al., 2017). In line with these findings, Huynh et al. (2014) reported a hypomethylated DNAme pattern for RARA in MS NAWM, which suggests no inflammatory activity (Huynh et al., 2014). Additionally, Marabita et al. (2017) found a smaller number of DNAme groups for RARA in tobacco smokers with MS compared to non-smokers diagnosed with MS, which is apparently related to tobacco consumption (Su et al., 2016). Cadherins are widespread calcium-dependent cell adhesion proteins involved in cell-cell adhesion, mobility, and proliferation of epithelial cells. In particular, cadherin 1 (CDH1) participates in many aspects of synapse formation and function (Almeida and Lyons, 2014). Recently, James et al. (2018) identified a large percentage of single nucleotide polymorphisms (SNPs) that may confer risk of MS. Among them, rs1886700 is statistically associated with lower expression of CDH1. Therefore, an increase of DNAme to CDH1 (Liggett et al., 2010; Huynh et al., 2014) probably leads to lower mRNA transcription rates and a worse disease course in MS (James et al., 2018).

## Conclusions and Perspectives

Multiple sclerosis is a chronic inflammatory CNS disease originating from a complex interaction between genes and the environment. As far as we know, genetics accounts for almost 30% of MS prevalence worldwide, while the remaining percentage corresponds to epigenetic modifications due to exposure to distinct environmental factors (Weber et al., 2007). This review summarized the common differentially methylated genes expressed in distinct cell types and tissues derived from MS patients in order to elucidate their role in the pathophysiology of the disease. We conclude that changes in the methylation status of certain genes cause chromosomal abnormalities, DNA damage, and the generation of pathogenic immune cells resulting in inadequate innate and adaptive immune response. Furthermore, we assume that aberrant epigenetic profile in the context of disease potentiates microgliosis, alterations in synapse formation and function, and a selective loss of GABAergic interneurons which in turn favors neurodegeneration. A deeper understanding of the underlying physiological mechanisms mediated by DNAme will contribute to the development of new strategies in MS prognosis and therapy.

## Author Contributions

NC and JT-R researched the literature and drafted the manuscript. JT-R critically reviewed and edited the work. Both authors read and approved the final manuscript.

## Funding

The author(s) disclosed receipt of the following financial support for the research, authorship, and/or publication of this article: This review was funded by the Deutsche Forschungsgemeinschaft to Dr. Jordi Tomas Roig (ref. TO 977/1-1) and the University of Girona to Mrs Naiara Celarain Sanz (ref. IFUdG2017).

## Conflict of Interest

The authors declare that the research was conducted in the absence of any commercial or financial relationships that could be construed as a potential conflict of interest.

## Abbreviations

MS, multiple sclerosis; CNS, central nervous system; APC, antigen presenting cells; Th, T helper cells; BBB, blood brain barrier; miRNA, microRNA; Me, methylation; Ub, ubiquitination; Ac, acetylation; P, phosphorylation; DNAme, DNA methylation; PBMCs, peripheral blood mononuclear cells; *AHR*, aryl hydrocarbon receptor; *AHRR*, aryl-hydrocarbon receptor repressor; *MHC*, major histocompatibility complex; *MICA*, MHC class I chain-related gene A; *MICB*, MHC class I chain-related gene B; NK, natural killer; *RUNX3*, runt related transcription factor 3; DC, dendritic cells; *CDKN2A*, cyclin dependent kinase inhibitor 2A; *GNAS*, guanine nucleotide binding protein alpha stimulating; IFN, type I interferon; *USP35*, ubiquitin specific peptidase 35; *ATP11A*, ATPase phospholipid transporting 11A; *TLR4*, toll-like receptor 4; pTh17, pathogenic Th17 cells; *RASA3*, RAS p21 protein activator 3; NAWM, normal appearing white matter; *HOXC4*, homeobox C4; *PTPRN2*, protein tyrosine phosphatase receptor type N2; *SMAD3*, SMAD family member 3; *TGFBI*, transforming growth factor beta induced; *SOCS1*, suppressor of cytokine signaling 1; ER, endoplasmic reticulum; PM, plasma membrane; TCRs, T-cell receptors; *MORN1*, MORN repeat containing 1; *PSD3*, pleckstrin and sec7 domain containing 3; *LINE-1*, long interspersed nuclear element; PTPs, protein tyrosine phosphatases; *KIF25*, kinesin family member 25; CAM, cell adhesion molecules; *IGSF9B*, immunoglobulin superfamily member 9B; *HLA-F*, HLA class I histocompatibility antigen alpha chain F; RA, retinoic acid; *RARA*, retinoic acid receptor alpha; *CDH1*, cadherin 1.
