# Supplementary material for: Changes in Deoxyribonucleic Acid Methylation Contribute to the Pathophysiology of Multiple Sclerosis
Source: Front Genet. 2019 Nov 12;10:1138. doi: 10.3389/fgene.2019.01138 (PMC6874160; doi:10.3389/fgene.2019.01138)
Supplement: Supplementary file 1 [file Table_1.docx]

| **Table S1. Changes in the DNA methylation of MS patients** | | |  |
| --- | --- | --- | --- |
| **References** | **Target sample** | **Differentially methylated genes** | |
|  |  |  | |
| [1] | CD8+ T cells | ***MS vs CTR:*** ERG, FTL, DCAF4, NCAPH2, CDKN1C, ZNF462, CBX7, MIR492, HPS1, SASH1, MYL3, KCNG1, DYDC2, MEGF10, SP5, LMO3, SLC12A7, MORN1, IGF2BP1, PLCB3, ABCC4, CREG2, CDC42BPB, UGT1A10, TMEM125, ARHGAP22, DACH1, OR8B12, TMEM8C, BAI1, EIF2S1, CRTAC1, DHX36, C19orf41, DLGAP2, TNXB, PRDM8, HEATR2, WHSC2, CAMTA1, ALK, KCNQ2, SCTR, RHEB, LOC202181, RRP9, KRT75, DGKE, PLD5, ZC3H14. | |
|  |  |  | |
| [2] | CD19+ B cells | ***RRMS vs CTR:*** LTA, SLC44A2, LTBR, CXCR5, CD19, IL21R, IL27, CARD11. | |
|  |  |  | |
| [3] | CD4+ T cells | ***RRMS vs CTR:*** MICA, MICB, HLA-DRB, HLA-DQB, MORN1, LCLAT1, PDCD1, MUC4, AHRR, ARSB, PCBD2, TGFBI, PCDHB13, PCDHB15, KIF25, CSGALNACT1, ADARB2, LDHAL6A, CORO1B, USP35, FUT4, ERC1, TCRA, PACS2, IL32, KCTD11, C17orf108, ARHGAP27, NPLOC4, SBNO2, GNG7, C21orf56, RIBC2. | |
|  |  |  | |
| [4] | Hippocampus | ***NAWM vs demyelinated MS brain:*** MLLT4, PPIF, SCRT2, SNRNP40, ISLR2, MEF2A, PMEPA1, ABCA4, ADAMTS12, AHRR, BEST3, CASP7, CCL4L2, CPXM2, FBXW8, HLA-B, LOC145845, MEIS1, MGMT, MYO7A, NXN, PKP2, PQLC1, PSD3, SCN4B, SDK2, SMYD3, TGFBI, TMEM165, PON1, HDLBP, MKKS, TRIM26, TRPS1, KRTAP27-1, MGP, AJAP1, C1orf106, C2orf62, DSE, EIF2C2, GATA5, HLA-B, IGSF9B, INSC, KIAA1026, KIF25, LOC100292680, NFASC, RASA3, SDK1, SHISA2, SOLH, SORBS2, TAGLN3, TBX5, TM9SF1, TOP1MT, ZSCAN1, AKNA, EBPL, FLJ42709, HERC6, OR52M1, SFRP1, C22orf43, LOC285830, NAPEPLD, NHLH2, PLCH1, SERPINA9, SLFN13, TMEM132B, TTLL3, WDR81. | |
|  |  |  | |
| [5] | PMBCs | ***RRMS vs CTR***: ASB2, ATP11A, CACNA2D3, CERS5, ESRRG, FRMD4A, GNAS, HOXC4-HOXC6, IFITM5, ILDR1, KCNK15, KLHL35, LEFTY2, PLEKHA2, RPH3AL, WRAP73, ZFYVE28.  ***PPMS vs CTR***: ATG16L2, CES1, CSGALNACT2, CYB5D1, LSMD1, FAM110A, GDF7, HKR1, HLA-F, HOXB13, IGSF9B, ILDR1, LDB2, MTPN, LUZP6, NTN1, OPCML, OR2L13, RBM46, TBX1, TCP10L, TMEM44, VIPR2, WRAP73.  ***RRMS vs SPMS***: ABCC5, AKAP12, CARS, CBFA2T3, CCDC67, FAM110A, FRMD4A, GIMAP5, HIVEP3, ICAM5, KCNQ1, KLF4, LEFTY2, OLFM3, PTH1R, RASA3, RNF39, RPH3AL, TRAF3, USP35, XKR5. | |
|  |  |  | |
| [6] | PMBCs | ***Tobacco smoker with MS vs non-smoker with MS:*** SRM, GNG12, GFI1, ANXA4, NFE2L2, ABLIM2, AHRR, SMIM3, CDKN1A, TPST1, CNTNAP2, SNTG1, MTSS1, PTK2, ZC3H3, ZMIZ1, PTGDR2, PRSS23, GRIK4, ETV6, RARG, LOC348021, CCDC88C, ITPK1, ANPEP, RARA, SMIM6, RECQL5, F2RL3, LINC00111, ACOT9. | |
|  |  |  | |
| [7] | NAWM | ***MS vs CTR:*** PAD2 | |
|  |  |  | |
| [8] | cfDNA (PBMCs) | ***RRMS vs CTR:*** MBP3, WM1. | |
|  |  |  | |
| [9] | NAWM | **MS vs CTR:** ALDOA, ATP1A2, BCAR1, BRK1, CDK5, CORO1A, CSF3, DLC1, DTNBP1, FGD2, FMNL1, MLST8, MYBPC3, MYH6, MYH7, MYO1F, OBSCN, PDGFA, PRKCZ, SHC1, SIPA1L1, SSH3, TPM3, ADA, AGAP1, ALDOA, ARHGEF16, ATP1A1, ATP1A2, ATP1A4, ATP5H, BIN1, DAB2IP, DLC1, FGD2, LDHC, MACROD1, MLST8, MYBPC3, MYH6, MYH7, NME4, NT5C, PLXNB1, PTPRN2, RASA3, SEPT9, SIPA1L1, TBCD, TK1, ACSBG1, ACSL1, ACTR8, ADA, AGAP1, AGPAT1, AGRP, AKAP8, ALDH3A1, ALDOA, AMH, ANGPT2, APBB1IP, APEX, ARHGEF16, ATF6B, ATP11A, ATP1A1, ATP1A2, ATP1A4, ATP6V0E1, ATRIP, BBS2, BCAR1, BCL2L2, BIN1, BIRC5, BPI, BRD4, BRK1, C4B, CACNA1D, CASKIN1, CBX4, CCL17, CCL22, CD37, CD59, CDH1, CDK5, CHST3, CHURC1, CLASP1, CLIC5, CORO1A, CREB5, CRY2, CSF3, CSNK1E, CX3CL1, CXXC5, CYP21A2, DAB2IP, DAND5, DCPS, DHRS3, DLC1, DLL1, DOK4, DOT1L, DSCAML1, DTNBP1, DYRK1B, E2F6, E4F1, EDN2, EFS, ENTPD2, ERCC3, F7, FAM109A, FGD2, FGFR3, FMNL1, GBX1, GDF10, GPR114, GPR56, GTF2H1, GYLTL1B, HDAC11, HEG1, HEXIM1, HEXIM2, HIGD1A, HIST3H3, HLA-DMA, IL17RB, IL25, IL34, INO80E, INPP5J, INTS1, IRAK2, ITPKB, JARID2, LIMD1, LMF1, LPCAT1, MAB21L2, MADD, MAML3, MAP3K14, MAPK3, MBP, MCF2L, MED24, MEIS2, MLLT10, MLST8, MT1A, MT1E, MT1F, MT1G, MT1M, MT2A, MT4, MTCH1, MTSS1L, MUSK, MYBPC3, MYH6, MYH7, MYO1F, NARFL, NCOR2, NDRG1, NLRP3, NOTCH4, NR1H3, NUP210, OBSCN, OTX2, PABPN1, PAG1, PBX2, PCSK6, PDGFA, PEG10, PHF21A, PIK3R1, PLEKHG3, PLLP, PLXNB1, POLD4, POLR2C, POU2F1, PPARA, PPIL2, PPP1R13B, PPP4C, PRAM1, PRDM16, PRKCH, PRKCZ, PTGDS, PTPRN2, RAD9A, RAI1, RASA3, RBP1, RFX5, RIN2, RNF187, RPA1, RRM2, RXRA, SACS, SEMA4C, SETD1A, SHC1, SHISA5, SIPA1L1, SLC17A7, SLC22A17, SLC39A13, SLC7A8, SMAD6, SOX1, SOX8, SPI1, SPOCK2, SREBF1, SSH3, SSTR5, SUN1, TACC3, TBCD, TBX6, TEAD2, TEF, TEP1, THRA, TLN2, TNRC6C, TPM3, TRAF2, TSNARE1, UBE2L3, USP19, VAC14, WHSC1, WISP1, WISP2, WNK2, ZBTB47, ZFP1, ZIC1, ZNF135, ZNF256, ZNF329, ZNF362, ZNF414, ZNF418, ZNF488, ZNF606, ZNF664, ZNF687, ADAMDEC1, AIF1, AIRE, B2M, BPI, C1QA, C1QB, C1QC, C4BPA, C4BPB, CCR6, CD19, CD37, CD4, CD7, CD81, CFD, DLG1, FCER2, HAMP, HLA-DMA, HLA-DMB, HLA-DOA, HLA-DOB, HLA-DQA2, HLA-DQB2, HLA-F, IRF6, IRF8, IRF9, JAK1, JAK3, KYNU, LAG3, LAT, LBP, LCP2, LGMN, LST1, LTA, LTB, MBL2, MICB, NCR3, OSM, PSMB8, PTPN22, RARA, RNF31, SECTM1, SLAMF7, STXBP2, TAP1, TAP2, TAPBP, TNF, TNIP2, B2M, C1QA, C1QB, C1QC, C4BPA, C4BPB, DLG1, FCER2, HLA-DMA, LAG3, LTA, MBL2, NCR3, SLAMF7, TAP1, TAP2, TNF, B2M, C1QA, C1QB, C1QC, C4BPA, C4BPB, DLG1, FCER2, HLA-DMA, LAG3, LAT, LTA, MBL2, NCR3, SLAMF7, STXBP2, TAP1, TAP2, TNF, B2M, FCER2, HAMP, LAG3, MBL2, NCR3, SLAMF7, STXBP2, TAP1, TAP2, BHLHE23, CTSZ, DLG1, DLL1, DLX1, DLX2, EDARADD, EPHB4, FOXL2, GLI1, GNAS, HOXC11, HOXC13, HOXC4, HOXC8, HOXC9, HOXD10, HOXD11, HOXD13, HOXD3, HOXD4, HOXD8, HOXD9, MSX1, PHLDA2, PPP1R13L, PTCD2, RARA, RUNX3, SOX1, SOX8, TBX3, TEAD2, TGM1, TH, TNF, TWIST1, WNT2, ZIC1. | |
|  |  |  | |
| [10] | CD4+ T cells  CD8+ T cells  Whole blood | ***RRMS vs CTR (CD4+T cells)****:* DCX, RDH13, DNHD1, TEKT5, TXNL1, MAGI2, TTC30B, APC2, TMEM48, ANGPTL2, RALGPS1, USP29, C20orf151, DLL1 6, DACH2, INPP5A, LOC727677, SEMA5B, SUGT1L1, HOXB2, OR10J5, RBMS1, C20orf151, AEN.  ***RRMS vs CTR (CD8+T cells):*** APC2, HOXA2, HRNBP3, HEXDC, NTRK3, DCX, TRIL, ARHGEF17, ESPNP, LHX5, TEKT5, LRRC43, CYP27C1, TMEM48, HHATL, AMMECR1, C19orf45, SRRM3, PSD3, PTPRN2, LOC654342, ARHGEF17, DNHD1, KIF1C, INCA1, VSIG1.  ***RRMS vs CTR (Whole blood):*** DACH2, LAMA2, TTLL8, GALNT9, POU3F4, NLRP12, PLS3, ANKRD1, CLSTN2, MAGEB4, APC2, PCDHA7, TMEM27, DNHD1, LGI1, PTCHD2, MMD2, HHATL, TMEM48, NXPH1, TDRD9, CDX1, YTHDC2, RGPD1, PLGLB2. | |
|  |  |  | |
| [11] | Buffy coat | ***RRMS vs PPMS vs SPMS vs CTR:*** SHP-1 | |
|  |  |  | |
| [12] | Whole blood  PBMCs  NAWM | ***MS vs CTR:*** IL2RA | |
|  |  |  | |
| [13] | PMBCs | ***MS treatment-naïve vs 1 year IFN-b vs CTR:*** LINE-1 | |
|  |  |  | |
| [14] | cfDNA (serum) | ***RRMS(e) vs RRMS (r) vs CTR:*** MOG | |
|  |  |  | |
| [15] | cfDNA (serum) | ***RRMS vs CTR:*** LINE-1 | |
|  |  |  | |
| [16] | T cells | ***RRMS vs CTR:*** VDR | |
|  |  |  | |
| [17] | cfpDNA | ***RRMS(r) vs CTR****:* CDH1, CDKN2A, CDKN2B, FAS, ICAM1, MCJ, MDGI, MUC2, MYF3, PAX5, PGK1, RB1, SOCS1, SYK, TP73.  ***RRMS(e) vs CTR****:* BRCA1, CCND2, DAPK, FAS, FHIT, MCT1, MDGI, MCJ, CDKN2A, TP73, PGK1, PR-PROX.  ***RRMS(r) vs RRMS(e)****:* CDH1, CDKN2B, HIC1, PR-PROX, SYK. | |
|  |  |  | |
| [18] | Whole Blood | ***RRMS(e) vs RRMS (r) vs CTR:*** CDKN2A, SOCS1, RUNX3, NEUROG1. | |
|  |  |  | |
| [19] | PMBCs  CD4+ T cells | ***Discordant twins (MS vs CTR):*** TMEM232, SEMA3C, YWHAGI, ZBTB16, MRI1, FIRRE. | |
|  |  |  | |
| [20] | PMBCs | ***RRMS and SPMS vs CTR:*** PAD2 | |
|  |  |  | |
| [21] | PMBCs | ***RRMS and SPMS vs CTR:*** DNMT1, TET2 | |
|  |  |  | |
| [22] | CD4+ T cells | ***RRMS and SPMS vs CTR:*** VMP1, MIR21 | |

MS: multiple sclerosis; CTR: control; RRMS: relapsing-remitting multiple sclerosis; PPMS: primary progressive multiple sclerosis; SPMS: secondary progressive multiple sclerosis; RRMS(e): RRMS in exacerbation;

RRMS (r): RRMS in remission; cfDNA: circulating-free DNA; cfpDNA: Cell free plasma DNA; PBMCs: peripheral blood mononuclear cells; NAWN: normal appearing white matter.

**Table S1 references:**

1. Maltby VE, Graves MC, Lea RA, Benton MC, Sanders KA, Tajouri L, et al. Genome-wide DNA methylation profiling of CD8+ T cells shows a distinct epigenetic signature to CD4+ T cells in multiple sclerosis patients. Clin Epigenetics. Clinical Epigenetics; 2015;7:1–6.
2. Maltby VE, Lea RA, Graves MC, Sanders KA, Benton MC, Tajouri L, et al. Genome-wide DNA methylation changes in CD19+ B cells from relapsing-remitting multiple sclerosis patients. Sci Rep. Springer US; 2018;8:17418.
3. Graves MC, Benton M, Lea RA, Boyle M, Tajouri L, Macartney-Coxson D, et al. Methylation differences at the HLA-DRB1 locus in CD4+ T-Cells are associated with multiple sclerosis. Mult Scler J. 2014;20:1033–41.
4. Chomyk AM, Volsko C, Tripathi A, Deckard SA, Trapp BD, Fox RJ, et al. DNA methylation in demyelinated multiple sclerosis hippocampus. Sci Rep. Springer US; 2017;7(1):8696.
5. Kulakova OG, Kabilov MR, Danilova L V., Popova E V., Baturina OA, Tsareva EY, et al. Whole-genome DNA methylation analysis of peripheral blood mononuclear cells in multiple sclerosis patients with different disease courses. Acta Naturae. 2016;8:103–10.
6. Marabita F, Almgren M, Sjöholm LK, Kular L, Liu Y, James T, et al. Smoking induces DNA methylation changes in Multiple Sclerosis patients with exposure-response relationship. Sci Rep. 2017;7:1–15.
7. Mastronardi FG, Noor A, Wood DD, Paton T, Moscarello MA. Peptidyl argininedeiminase 2 CpG island in multiple sclerosis white matter is hypomethylated. J Neurosci Res. 2007;85(9):2006-16.
8. Lehmann-Werman R, Neiman D, Zemmour H, Moss J, Magenheim J, Vaknin-Dembinsky A, et al. Identification of tissue-specific cell death using methylation patterns of circulating DNA. Proc Natl Acad Sci. 2016;113:E1826–34.
9. Huynh JL, Garg P, Thin TH, Yoo S, Dutta R, Trapp BD, et al. Epigenome-wide differences in pathology-free regions of multiple sclerosis-affected brains. Nat Neurosci. Nature Publishing Group; 2014;17:121–30.
10. Bos SD, Page CM, Andreassen BK, Elboudwarej E, Gustavsen MW, Briggs F, et al. Genome-wide DNA methylation profiles indicate CD8+ T cell hypermethylation in multiple sclerosis. PLoS One. 2015;10:1–16.
11. Kumagai C, Kalman B, Middleton FA, Vyshkina T, Massa PT. Increased promoter methylation of the immune regulatory gene SHP-1 in leukocytes of multiple sclerosis subjects. J Neuroimmunol. Elsevier B.V.; 2012;246:51–7.
12. Field J, Fox A, Jordan MA, Baxter AG, Spelman T, Gresle M, et al. Interleukin-2 receptor-α proximal promoter hypomethylation is associated with multiple sclerosis. Genes Immun. Nature Publishing Group; 2017;18:59–66.
13. Pinto-Medel MJ, Oliver-Martos B, Urbaneja-Romero P, Hurtado-Guerrero I, Ortega-Pinazo J, Serrano-Castro P, et al. Global methylation correlates with clinical status in multiple sclerosis patients in the first year of IFNbeta treatment. Sci Rep. 2017;7:1–9.
14. Olsen JA, Kenna LA, Tipon RC, Spelios MG, Stecker MM, Akirav EM. A Minimally-invasive Blood-derived Biomarker of Oligodendrocyte Cell-loss in Multiple Sclerosis. EBioMedicine. The Authors; 2016;10:227–35.
15. Dunaeva M, Derksen M, Pruijn GJM. LINE-1 Hypermethylation in Serum Cell-Free DNA of Relapsing Remitting Multiple Sclerosis Patients. Mol Neurobiol. Molecular Neurobiology; 2018;55:4681–8.
16. Ayuso T, Aznar P, Soriano L, Olaskoaga A, Roldán M, Otano M, et al. Vitamin D receptor gene is epigenetically altered and transcriptionally up-regulated in multiple sclerosis. PLoS One. 2017;12:1–10.
17. Liggett T, Melnikov A, Tilwalli S, Yi Q, Chen H, Replogle C, et al. Methylation patterns of cell-free plasma DNA in relapsing-remitting multiple sclerosis. J Neurol Sci. Elsevier B.V.; 2010;290:16–21.
18. Sokratous M, Dardiotis E, Bellou E, Tsouris Z, Michalopoulou A, Dardioti M, et al. CpG Island Methylation Patterns in Relapsing-Remitting Multiple Sclerosis. J Mol Neurosci. Journal of Molecular Neuroscience; 2018;64:478–84.
19. Souren NY, Gerdes LA, Lutsik P, Gasparoni G, Beltran E, Salhab A, et al. DNA methylation signatures of a large cohort monozygotic twins clinically discordant for multiple sclerosis. bioRxiv. 2018.
20. Calabrese R, Zampieri M, Mechelli R, Annibali V, Guastafierro T, Ciccarone F, et al. Methylation-dependent PAD2 upregulation in multiple sclerosis peripheral blood. Mult Scler J. 2012;18:299–304.
21. Calabrese R, Valentini E, Ciccarone F, Guastafierro T, Bacalini MG, Ricigliano VAG, et al. TET2 gene expression and 5-hydroxymethylcytosine level in multiple sclerosis peripheral blood cells. Biochimica et biophysica acta. 2014;1842(7):1130–6.
22. Ruhrmann S, Ewing E, Piket E, Kular L, Cetrulo Lorenzi JC, Fernandes SJ, et al. Hypermethylation of MIR21 in CD4+ T cells from patients with relapsing-remitting multiple sclerosis associates with lower miRNA-21 levels and concomitant up-regulation of its target genes. Mult Scler J. 2017;24(10):1288-1300.
